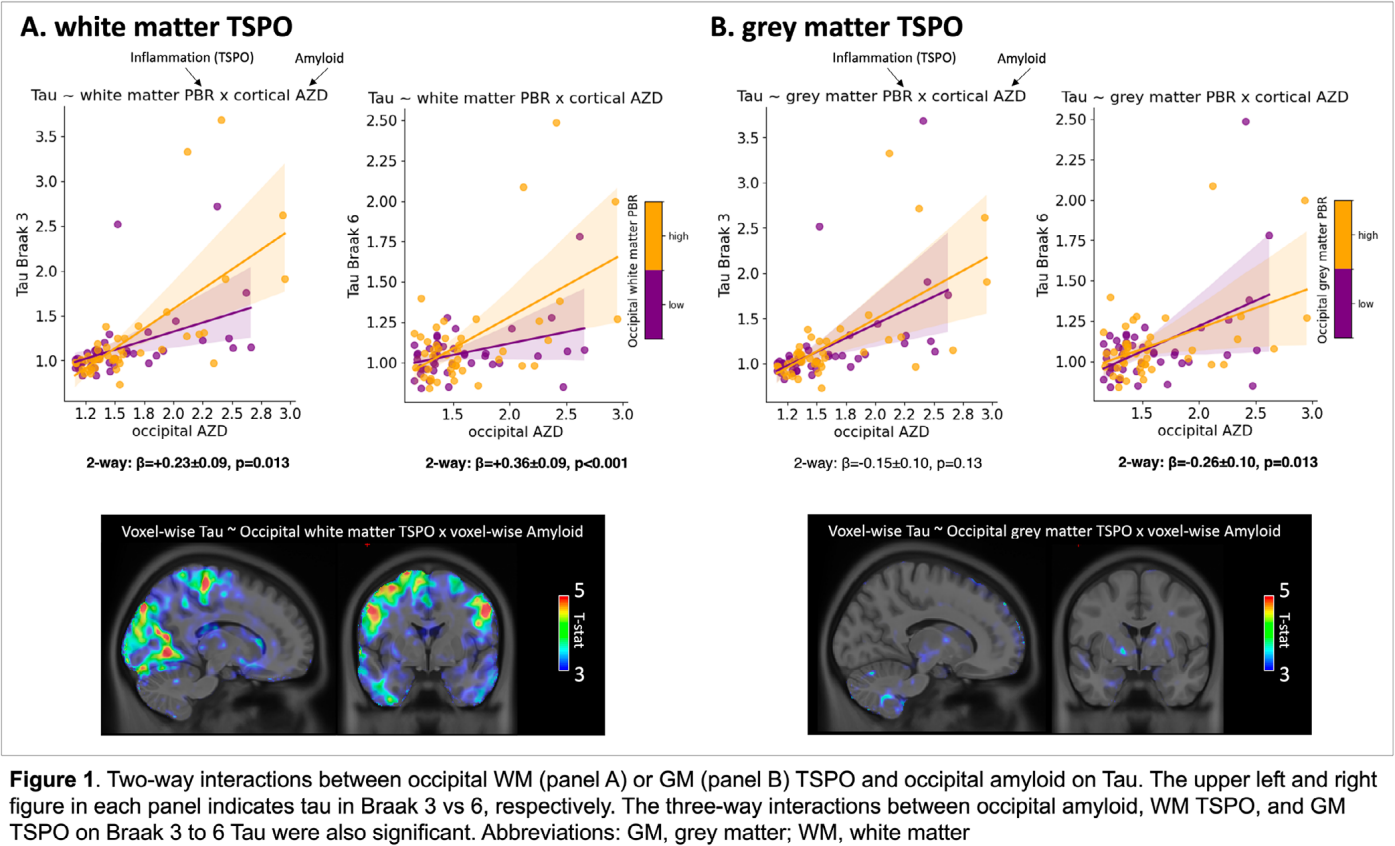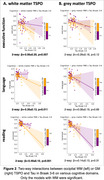# Glial inflammation in the posterior white matter modulates the amyloid‐tau‐cognition relationship

**DOI:** 10.1002/alz70856_098210

**Published:** 2025-12-24

**Authors:** Julie Ottoy, Min Su Kang, Eric Yin, Nesrine Rahmouni, Jenna Stevenson, Gleb Bezgin, Jean‐Paul Soucy, Andrea Benedet, Kaj Blennow, Thomas K Karikari, Henrik Zetterberg, Nicholas J. Ashton, Serge Gauthier, Sandra E. Black, Pedro Rosa‐Neto, Maged Goubran

**Affiliations:** ^1^ LC Campbell Cognitive Neurology Research Unit, Sunnybrook Research Institute, University of Toronto, Toronto, ON, Canada; ^2^ Dr. Sandra E. Black Centre for Brain Resilience and Recovery, LC Campbell Cognitive Neurology, Hurvitz Brain Sciences Program, Sunnybrook Research Institute, University of Toronto, Toronto, ON, Canada; ^3^ Translational Neuroimaging Laboratory, The McGill University Research Centre for Studies in Aging, Montréal, QC, Canada; ^4^ Montreal Neurological Institute, McGill University, Montreal, QC, Canada; ^5^ Institute of Neuroscienace and Physiology, University of Gothenburg, Mölndal, Västra Götaland, Sweden; ^6^ Clinical Neurochemistry Laboratory, Sahlgrenska University Hospital, Mölndal, Sweden; ^7^ Department of Psychiatry and Neurochemistry, University of Gothenburg, Gothenburg, Sweden; ^8^ University of Pittsburgh, Pittsburgh, PA, USA; ^9^ Department of Neurodegenerative Disease, UCL Queen Square Institute of Neurology, University College London, London, ‐, United Kingdom; ^10^ Institute of Neuroscience and Physiology, Sahlgrenska Academy at the University of Gothenburg, Gothenburg, Sweden; ^11^ UK Dementia Research Institute at UCL, London, United Kingdom; ^12^ Banner Alzheimer's Institute, Phoenix, AZ, USA; ^13^ McConnell Brain Imaging Centre, Montreal Neurological Institute, McGill University, Montreal, QC, Canada; ^14^ Department of Medical Biophysics, University of Toronto, Toronto, ON, Canada

## Abstract

**Background:**

White matter inflammation significantly contributes to the progression of Alzheimer's disease (AD). Changes in the translocator protein (TSPO), an in‐vivo marker of brain inflammation, have been observed in white matter (WM) through in‐vitro binding assays, immunostaining, and TSPO PET‐based quantification. Our objective was to assess the effects of glial inflammation in WM via TSPO‐PET on AD biomarkers and cognitive decline, beyond grey matter (GM) inflammation, across the AD spectrum.

**Method:**

Eighty‐eight elderly participants from the Translational Biomarkers in Aging and Dementia cohort (44% Aβ‐positive, 38% cognitively impaired) underwent diffusion‐weighted MRI, TSPO‐PET (^11^C‐PBR28), amyloid‐PET (^18^F‐AZD4694), tau‐PET (^18^F‐MK6240), and plasma Aβ42/40, ptau181, ptau231, GFAP, and NfL. Diffusion‐weighted modeling differentiated free water from fiber integrity indexed by fractional anisotropy. Inflammation was assessed via TSPO standardized uptake value ratios normalized to cerebellar grey. We investigated associations of the TSPO signal in the normal‐appearing WM (eroded by 2mm^3^) with each of the AD biomarkers, diffusion metrics, and cognition; adjusted for age, sex, (and education). GM and WM TSPO signals were tested in the same models.

**Result:**

We observed a significant three‐way interaction between amyloid, GM TSPO, and WM TSPO on Tau (Braak‐3 tau: *p* = 0.013, Braak‐4: *p* = 0.029, Braak‐5: *p* = 0.018, Braak‐6: *p* = 0.005). Decomposing this interaction, individuals with greater amyloid in the presence of greater occipital WM TSPO displayed greater Tau (Figure 1A, left: Braak‐3 tau, right: Braak‐6 tau). No such effect was found with GM TSPO in the occipital lobe (Figure 1B) but there was a significant effect with GM TSPO in the cingulate cortex (Braak‐45 tau: *p* = 0.001‐0.004). Further, TSPO signal in the occipital WM was associated with greater concentrations of plasma GFAP (*p* = 0.039) and with lower fiber integrity (*p* = 0.029), but there was no association with plasma Aβ4240, ptau231, ptau181, or NfL. Last, TSPO signal in the occipital WM modulated the effect of amyloid‐related tau increases on several cognitive domains (including executive function and language; Figure 2).

**Conclusion:**

Inflammation in the posterior WM, as quantified through TSPO‐PET, modulated the relationship between amyloid and tau and between amyloid‐related tau effects on cognitive dysfunction. These effects were beyond GM TSPO. Our findings highlight the importance of WM inflammation in the pathogenesis of AD.